# 730 Geographic Effects of Rural Burn Care Follow Up During the COVID-19 Pandemic

**DOI:** 10.1093/jbcr/irad045.205

**Published:** 2023-05-15

**Authors:** Evan Bradshaw, Liza Garcia, Catherine Anding, Alan Pang, John Griswold, Deepak Bharadia

**Affiliations:** Texas Tech University Health Sciences Center, Amarillo, Texas; Texas Tech University Health Sciences Center, Lubbock, Texas; Texas Tech University Health Sciences Center, Lubbock, Texas; Texas Tech University Health Sciences Center, Lubbock, Texas; Texas Tech University Health Sciences Center, Lubbock, Texas; Texas Tech University Health Sciences Center, Lubbock, Texas; Texas Tech University Health Sciences Center, Amarillo, Texas; Texas Tech University Health Sciences Center, Lubbock, Texas; Texas Tech University Health Sciences Center, Lubbock, Texas; Texas Tech University Health Sciences Center, Lubbock, Texas; Texas Tech University Health Sciences Center, Lubbock, Texas; Texas Tech University Health Sciences Center, Lubbock, Texas; Texas Tech University Health Sciences Center, Amarillo, Texas; Texas Tech University Health Sciences Center, Lubbock, Texas; Texas Tech University Health Sciences Center, Lubbock, Texas; Texas Tech University Health Sciences Center, Lubbock, Texas; Texas Tech University Health Sciences Center, Lubbock, Texas; Texas Tech University Health Sciences Center, Lubbock, Texas; Texas Tech University Health Sciences Center, Amarillo, Texas; Texas Tech University Health Sciences Center, Lubbock, Texas; Texas Tech University Health Sciences Center, Lubbock, Texas; Texas Tech University Health Sciences Center, Lubbock, Texas; Texas Tech University Health Sciences Center, Lubbock, Texas; Texas Tech University Health Sciences Center, Lubbock, Texas; Texas Tech University Health Sciences Center, Amarillo, Texas; Texas Tech University Health Sciences Center, Lubbock, Texas; Texas Tech University Health Sciences Center, Lubbock, Texas; Texas Tech University Health Sciences Center, Lubbock, Texas; Texas Tech University Health Sciences Center, Lubbock, Texas; Texas Tech University Health Sciences Center, Lubbock, Texas; Texas Tech University Health Sciences Center, Amarillo, Texas; Texas Tech University Health Sciences Center, Lubbock, Texas; Texas Tech University Health Sciences Center, Lubbock, Texas; Texas Tech University Health Sciences Center, Lubbock, Texas; Texas Tech University Health Sciences Center, Lubbock, Texas; Texas Tech University Health Sciences Center, Lubbock, Texas

## Abstract

**Introduction:**

Follow-up appointments (FUAs) are crucial to monitor proper healing in burn patients. This study aims to determine if there is a predictive relationship between the distance a patient lives from the burn center and FUA attendance This study also aims to determine if the COVID-19 pandemic had any effect on FUA attendance at our burn center.

**Methods:**

Data was collected from the Burn Registry of a rural, ABA verified Regional Burn Center on patients who were admitted for an inpatient stay and discharged from January, 2019 through December, 2020. For each patient, we recorded demographic factors, home zip code, TBSA, length of inpatient stay, and FUA outcome. We split the resulting cohort in two separate ways by FUA status and pandemic status based on if they did/did not attend followup and were admitted before 3/9/20 (pre-pandemic) or after 3/9/20 (intra-pandemic). Chi Square Tests were used to determine if the rate of followup differed across pandemic status. We then ran Mann-Whitney U Tests to compare all the variables across both pandemic status and FUA status.

**Results:**

The cohort consisted of 511 patients. The pre-pandemic followup rate was 71.0% (n = 334) and the intra-pandemic followup rate was 71.2% (n = 177). This was not a statistically significant difference (*Χ*^2^ = 0.003, df = 1, p = 0.96). The next analysis compared the variables across pandemic status. The only significantly different variable between the groups was an increased percentage of 2nd degree burns in the intra-pandemic group (U = 24656.0, *p* = 0.018). The next analysis compared the variables across FUA status. Age, distance to the burn clinic, and amount of 3rd degree burns significantly differed across FUA status groups (U = 22789.0, *p* = 0.007; U = 19946.5, *p* = 0.000; U = 22738.5, *p* = 0.037, respectively), indicating the group that did follow up was younger, lived closer, and had more 3rd degree burns.

**Conclusions:**

Furthermore, the results indicate that the distance a patient lives from our center is the highest predictive variable in determining whether or not a patient will attend followup.

**Applicability of Research to Practice:**

This research is especially applicable to practitioners in rural environments or those evaluating the reasons for lack of follow-up in burn care.

